# Association of Automated Text Messaging With Patient Response Rate After Same-Day Surgery

**DOI:** 10.1001/jamanetworkopen.2020.33312

**Published:** 2021-01-13

**Authors:** Marie-Laure Cittanova, Sophie Chauvier, Evelyne Combettes, Nicolas Boccheciampe, Vincent Gerbier, Marc Leone, Jean-Michel Constantin

**Affiliations:** 1 Clinique Saint Jean de Dieu, Department of Anesthesiology, Fondation Cognacq Jay, Paris, France; 2 Chauvier Ambulatory unit, Clinique Saint Jean de Dieu, Fondation Cognacq Jay, Paris, France; 3 Nord Hospital Department of Anesthesiology and Critical Care, Assistance Publique Hôpitaux Universitaires de Marseille, Aix Marseille Université, Marseille, France; 4 Department of Anesthesiology and Critical Care, Pitié-Salpêtrière Hospital, Sorbonne University, Paris, France

## Abstract

This quality improvement study assesses the association of automated text messaging follow-up with the patient response rate after same-day surgery.

## Introduction

After same-day surgery, a telephone call (TC) for patient follow-up is recommended the next day.^1,2^ However, most TCs do not result in interventions, and there is a high rate of no response from patients.^3,4^ Therefore, we sought to evaluate postoperative recovery using an automated text message (TM) system (Memoquest; Calmedica).^5^

## Methods

This single-center, before-after, quality improvement study consists of 3 periods: the first period (May 1, 2016, through April 30, 2017), a 2-month washout period, and a second period (July 1, 2017, through June 30, 2018). The study population consisted of consecutive adult patients admitted for same-day surgery. All patients were admitted to Saint Jean de Dieu Clinic, Paris, France. The French Society of Anesthesiology and Intensive Care Medicine Ethics Committee (CERAR IRB 00010254) approved the study and granted an informed consent waiver. French law on biomedical research does not apply to this study because it is routine practice. The study followed the Strengthening the Reporting of Observational Studies in Epidemiology (STROBE) reporting guideline.

During the first period, all patients undergoing same-day surgery (except cataract surgery procedures) were contacted by telephone the next day for follow-up. During the second period, nurses verbally told patients that they would be contacted the next day via an automated TM sent by Memoquest and provided them with printed patient information outlining the TM procedure (eFigure in the Supplement).^4,5^

The primary study end point was the rate of patients successfully reached by either follow-up method. The secondary end points were patient satisfaction, nurse satisfaction (5 nurses responded to the questionnaire), and cost-effectiveness. Satisfaction was assessed using a 5-point Likert scale, for which 1 indicated unsatisfied and 5 indicated fully satisfied. The costs of each follow-up method were also computed (eMethods in the Supplement).

Data were analyzed from January 7 to June 28, 2019. For group comparison, a 2-tailed unpaired *t* test and parametric χ^2^ test were used as appropriate. Analyses were performed with Stata, version 13.0 software (StataCorp LP). A 2-sided *P* > .05 was considered to be statistically significant.

## Results

The patient demographic characteristics were similar in both groups. The TC group consisted of 8134 patients (3254 [40%] men and 4880 [60%] women), with a mean (SD) age of 58 (23) years. The TM group included 9325 individuals (3637 [39%] men and 5688 [61%] women), with a mean (SD) age of 56 (23) years. The primary end point was reached for 2805 (49.2%) patients in the TC group and 7954 (85.3%) patients in the automated TM group (*P* < .001).

In the TC group of 8134 patients, 2428 were excluded (1924 [79.2%] who had cataract surgery and 504 [20.8%] lost to follow-up), leaving a total of 5706 patients contacted by TCs. Of these, 2901 (50.8%) did not respond or call the hospital back. Of the remaining 2805 respondents, 2606 (92.9%) reported that “Everything is fine” and 199 (7.1%) reported complications. Complications were pain level higher than 3 on a scale of 0 to 10 (0 indicated no pain and 10 indicated worst imaginable pain) and medication taken, nausea or vomiting, bleeding, or fever.

In the TM group of 9325 patients, 921 were unable to receive automated TMs; 302 of these patients were ultimately contacted by TCs. Of the 8404 patients who received the first automated TM, 6903 respondents (82.1%) reported “Everything is fine,” 192 respondents (2.3%) reported that everything is fine but in an unexpected format (ie, typing errors, comments about the stay), and 255 (3.0%) required a telephone call for complications. Of the 1054 patients (12.6%) who did not respond to the automated TM or responded late, these patients were called by telephone and 699 (8.3%) were lost to follow-up (Figure).

**Figure.**
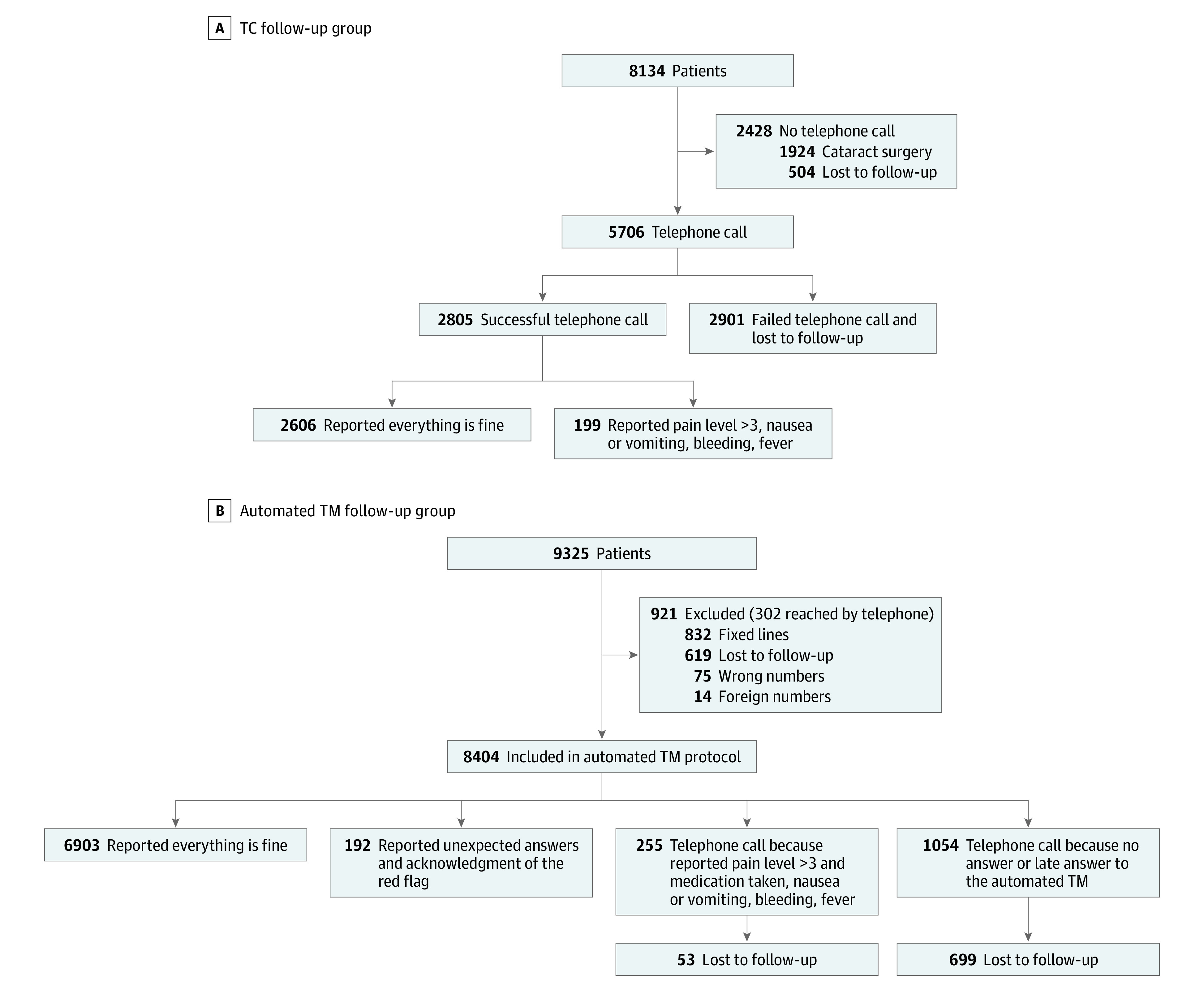
Flowchart of Follow-up Methods Pain scores were rated 0 through 10, with 0 indicating no pain and 10 indicating the worst imaginable pain. The complication rate may differ between the 2 groups because cataract surgery was excluded in the telephone call (TC) period because it is not a painful surgery. In the TM group, ultimately, 1371 patients were lost to follow-up, and 7954 patients were successfully reached by automated text messages (TMs). The 1371 patients lost to follow-up included 619 patients unable to receive automated TMs and not reached by telephone, 699 patients who did not respond to the automated TM and not reached by telephone, plus 53 patients called because of suspected complications who did not respond. Unexpected responses could be typing errors not detected by the computer system, comments about the stay, the hospital, or other entry. In these cases, the nurse could manually acknowledge the red flag. All other warnings led to a phone call by the nurse.

During both periods, patients in both groups were 99% satisfied or fully satisfied (*P* = .92), with a satisfaction score in the automated TM group of 4.7 points on the 5-point Likert scale. Meanwhile, nurse satisfaction (n = 5) increased between the 2 periods (eg, from a mean [SD] score of 1.8 [1.8] for ease of implementation in the TC group to 5.0 [0.0] in the automated TM group; *P* < .001) (Table).

**Table.** Satisfaction With Follow-up Methods Among the 5 Nurses in the Study

Variable	Mean (SD) score^a^	
	Telephone call group	Automated text message group
Ease of implementation	1.8 (1.8)	5.0 (0.0)^b^
Time gain	1.0 (0.0)	5.0 (0.0)^b^
Work interest	1.0 (0.0)	4.8 (0.5)^b^
Call relevance	1.0 (0.0)	5.0 (0.0)^b^
User-friendliness	1.0 (0.0)	4.8 (0.5)^b^

^a^ Nurse satisfaction with the automated test message system was compared with the next-day telephone call using a 5-point Likert satisfaction scale ranging from 1 (unsatisfied) to 5 (fully satisfied).

^b^ *P* < .001 for comparison of the follow-up methods.

The total follow-up cost in the TC group was estimated at $25 413. For the 5706 patients who were called, for reasons of availability, the follow-up cost was at $17 830. In the automated TM group, the follow-up cost was $17 597 for 8404 patients. The follow-up cost per patient was $2.09 in the automated TM group compared with $3.12 for the TC group.

## Discussion

In this quality improvement study, the use of automated TM implementation was associated with improved patient follow-up and nurse satisfaction. The automated TM system had multiple strengths. First, mobile telephones, and not smartphones, were required. This was helpful because, in France, the penetration of smartphone decreases with age, which would result in disparities for elderly patients.^6^^(pp30,35)^ Second, an active role of the patients was not required. Third, they received the automated TM regardless of their location. Finally, the process was cost-effective.

The use of automated TMs was associated with a lower number of patients lost to follow-up. As in previous studies,^4,5^ the loss was less than 10% in the automated TM group compared with 50% in the TC group. This study had some limitations. First, the design was a single-center, before-after, quality improvement study. Second, when a complication occurred, there was a low rate of appropriate responses to the automated TM questionnaire. If there was a complication, a health professional had to call the patient. Third, long-term outcomes were not assessed. Fourth, the nurses who no longer work in the same-day surgery unit may have had different levels of satisfaction. Fifth, a future implementation of an automated TM program could introduce disparities due to differences in familiarity and use of text messaging. In conclusion, the shift from TCs to the automated TM system was associated with an increase in successful follow-up, nurse satisfaction, and cost-effectiveness.

## Notes

Supplement. **eMethods.** Supplementary Methods **eFigure.** Patient Information
