# Supplementary material for: Association of Automated Text Messaging With Patient Response Rate After Same-Day Surgery
Source: JAMA Netw Open. 2021 Jan 13;4(1):e2033312. doi: 10.1001/jamanetworkopen.2020.33312 (PMC7807290; doi:10.1001/jamanetworkopen.2020.33312)
Supplement: Supplement. — eMethods. Supplementary Methods eFigure. Patient Information [file jamanetwopen-e2033312-s001.pdf]

Supplemental Online Content

Cittanova ML, Chauvier S, Combettes E, et al. Association of automated text messaging with patient response rate after same-day surgery. *JAMA Netw Open.* 2021;4(1):e2033312. doi:10.1001/jamanetworkopen.2020.33312

eMethods. Supplementary Methods

eFigure. Patient Information

This supplementary material has been provided by the authors to give readers additional information about their work.

© 2021 Cittanova ML et al. *JAMA Network Open.*

eMethods. Supplementary Methods

The study adheres to STROBE statements. As that was an exploratory study, the number of patients was determined by the number of patients undergoing day surgery during the study period.

Procedure

At 11:30 a.m., in the absence of a response or on receipt of a satisfactory one, i.e. “Everything is fine”, the patient received two other automated TMs, measuring pain and other potential complications, such as nausea/vomiting, fever, or major bleeding. If the patient responded “pain”, she/he received a new automated TM, questioning her/him about the pain intensity. The patients had been educated about the 10-point numerical pain scale during their stay in the day surgery unit, where the pain scale level was used several times and explained by healthcare professionals. If the level of pain was above 3, the patient was asked about taking pain medication. If the response was “taken”, or if the patient had other complications, such as nausea/vomiting, major bleeding, or fever, a red flag consisting of the word “alert” in red flashed on the computer to prompt a phone call by a nurse. The whole TM conversation was logged in the patient’s computerized medical file. If the alert corresponded to an unexpected response, such as typing errors that were not detected by the computer system, or comments about the stay, the hospital, or something else, but without mentioning any complication, or if the patients had a pain level equal to or below 3, the nurse could manually acknowledge the alarm. All other warnings led to a phone call by the nurse.

© 2021 Cittanova ML et al. *JAMA Network Open.*

Cost calculation

The costs were computed as follows. The phone call duration was estimated at 6 minutes, with an hourly salary of a nurse costed at $31.24 (exchange rate: €1 is equivalent to $1.18). The cost of the automated TM system was divided into follow-up and satisfaction. For postoperative follow-up, the initial costs were $711 to set up the system, and the connection with the patient's computerized file had a cost evaluated at a one-off cost of $2820. We considered that this investment would be amortized over five years, thus representing $706 yearly depreciation expenses. Annual costs for follow-up included $853 fixed costs plus $1.42 per patient. For satisfaction, the annual costs were calculated as $706 depreciation expenses, $853 fixed costs, and $0.71 per patient.

© 2021 Cittanova ML et al. *JAMA Network Open.*

eFigure. Patient Information

CLINIQUE SAINT JEAN DE DIEU
GCS Oudinot - Cognacq-Jay

Let's stay in touch……. tomorrow by TM !!

✓ **Tomorrow, around 10:30am, we shall contact you in order to have some news about you. It will be by TM.**

In order to avoid multiple calls to telephones and getting answering machines, we have chosen to use TM messages. We believe it is the right system if we want to be reassured about your health and wellbeing. It will also help us to answer quickly and adequately any questions you may have.

✓ **Will the TM replace the contact on the telephone? No !**

The nurses will contact you directly in case of any need (pain, fever or other). The ambulatory service is reachable at 01 40 61 46 44. The clinic reception is available 24/7 at **01 40 61 11 00.**

✓ **You will also receive a satisfaction questionnaire on your phone as a TM.**

You are invited to answer it by a notation from 1 to 5, with 5 being the best satisfactory grade.

1 TM the day after if you are in day surgery

Saint Jean de Dieu Clinic (01 40 61 46 44) : "Hello, as part of your follow-up after surgery, if everything is fine with you, please answer EIF (everything is fine)

→ If your answer is "**EIF**", then we shall be glad to know that you are fine.

2 In case of a non-response or something other than "EIF", you will receive the following TM :

If you experience nausea, vomiting, fever over 38°C, or important bleeding, please answer YES.

→ Answer **YES** or **NO**.
If YES is your answer, we shall call you as soon as possible.

+

Evaluate your pain on a self-evaluation scale from 0 to 10. Please use only one number. (0: no pain, 10: unbearable pain)

→ Please use only one number. (0: no pain, 10: unbearable pain)
If your pain is over 3, this will lead to another TM from the clinic

If you have taken painkillers, answer 'TAKEN", otherwise take them now and send your new pain evaluation by TM after 45 minutes.

→ ⇒ Answer "TAKEN" if you are still in pain after having taken your painkillers.
⇒ If you have not yet taken your painkillers, take them, wait 45 minutes and reset your pain evaluation (0 to 10)

© 2021 Cittanova ML et al. *JAMA Network Open.*
